# Effects of Dairy Matrix on the Intestinal, Liver, and Bone Transcriptome of Healthy Rats

**DOI:** 10.3390/foods14081375

**Published:** 2025-04-16

**Authors:** Xiaorui Zhao, Martin Krøyer Rasmussen, Axel Kornerup Hansen, Hanne Christine Bertram

**Affiliations:** 1 Department of Food Science, Aarhus University, Agro Food Park 48, DK-8200 Aarhus N, Denmark; xiaorui@food.ku.dk (X.Z.); martink.rasmussen@food.au.dk (M.K.R.); 2 Department of Veterinary and Animal Sciences, University of Copenhagen, Ridebanevej 9, DK-1870 Frederiksberg C, Denmark; akh@sund.ku.dk

**Keywords:** fermented dairy, inulin, gut–organ axes, transcriptomics

## Abstract

Fermentation is one of the oldest food processing techniques and is widely utilized in dairy product processing, during which nutrient availability and bioactive compounds are altered. However, the complete mode of action by which fermented dairy exerts beneficial effects on the host remains unknown. The present study investigated the effect of milk and yogurt ingestion alone or combined with prebiotic inulin on the transcriptome of colonic mucosa, liver, and femur in healthy rats. Young growing male rats were fed one of four experimental diets containing (1) skimmed milk, (2) skimmed milk supplemented with inulin (5% *w*/*w*), (3) yogurt, or (4) yogurt supplemented with inulin (5% *w*/*w*) for 6 weeks. Microarray results revealed that yogurt consumption resulted in 2195 upregulated differential expressed genes (DEGs) and 1474 downregulated DEGs in colonic mucosa as compared with milk consumption. According to Gene Ontology (GO) categories and Kyoto Encyclopedia of Genes and Genomes (KEGG) pathway analysis, tight junction-, immune system-related pathways in the colonic mucosa and metabolic pathways in the liver were enriched with yogurt consumption. No evident differences were identified in the bone transcriptome between the diet groups. In conclusion, the study found that the intake of fermented dairy exerts more pronounced effects on gene expression in the intestinal tissue than prebiotics supplementation.

## 1. Introduction

Dairy products are nutrient-dense foods containing high-quality protein and essential micronutrients, which constitute an important part of the habitual diet for many people around the world [1,2]. In addition to supplying calcium to benefit bone health, dairy product consumption may also influence the gut milieu favorably by modulating the gut metabolome [2] and the intestinal barrier [3]. Epidemiological data also show that dairy product consumption reduces the risk of certain liver diseases, such as non-alcoholic fatty liver disease [4] and liver cancer [5].

As one of the oldest food processing techniques, fermentation has been utilized widely in dairy product processing. Thus, milk is used for the production of yogurt via bacterial fermentation during which complex organic compounds are broken down and undergo biochemical transformations, and nutrient availability and bioactive compounds are altered [6]. In addition, compared to unfermented forms (milk), fermented dairy products such as yogurt contain beneficial bacteria added as a starter culture, mainly including *Streptococcus thermophilus* and *Lactobacillus delbrueckii*, which have been proposed to exert a potential regulatory effect on mucosal immunity and on the function of the intestinal barrier [7].

Prebiotics, such as the plant fiber inulin, are considered to impact the host health mainly via modulating the gut milieu, and the biofunctional benefits of combining dairy products and prebiotics is gaining interest. Some studies reported that the addition of inulin to yogurt increased fecal bifidobacteria [8,9]; similarly, the supplementation of inulin-type fructans in cheese also led to changes in gut microbiome upon ingestion [10].

The transcriptome represents the complete set of RNA transcripts in a specific cell type or tissue at a certain developmental stage and/or under a specific physiological condition. Thus, transcriptomics focuses on gene expression at the RNA level, provides genome-wide information on gene structure and gene function, and further unveils the regulation network of biological processes [11]. In recent years, transcriptomics has emerged as a useful tool to decipher how diets may induce transcriptional changes. Thus, by applying transcriptomics, it was demonstrated in rats how a Western diet-induced rapid liver gene expression changes [12] and that dietary protein levels impacted the epithelial transcriptome [13]. In a previous rat study, transcriptomics enabled us to identify that inulin-fortification of milk enhanced ferritin content in the liver compared to milk diet [14]. The study thereby demonstrated how transcriptomics can be an effective tool for elucidating gut–organ axes that have been proposed [15]. Thus, in the current study, we used transcriptomics as the main tool to probe diet-induced gut–organs interactions at the molecular level.

The study aimed to test the hypotheses that (i) the intake of fermented dairy influences the colonic mucosa through effects at the transcriptional level, (ii) supplementation with prebiotic inulin will augment these transcriptional changes, and (iii) diet-induced gut–liver and gut–bone axes involve effects at the transcriptional level. To test these hypotheses, we investigated how milk and yogurt alone and in combination with inulin influence the colonic mucosa, liver, and bone transcriptome to decipher potential gut–organ axes. To achieve this, a 6-week diet intervention study was conducted in young, growing male rats, and the colonic mucosa scrubs (CMS), liver, and femur bone were collected for transcriptomic profiling and real-time qPCR determination.

## 2. Materials and Methods

### 2.1. Rat Intervention Study

The rat intervention study was conducted in accordance with Directive 2010/63/EU on animal ethics and welfare and the Danish Animal Experimentation Act (LBK 1107/01-07-2022). The study was approved by the Animal Experiments Inspectorate in Denmark (License No 2020-15-0201-00434) and conducted at the Department of Veterinary and Animal Sciences at Copenhagen University. Details on the animals and experimental diet intervention performed for the study have been previously described [16]. In brief, male Sprague Dawley rats (6 weeks of age, body weight approx. 200 g) were randomly assigned to one of four diet groups: Milk (50% *w*/*w* skimmed milk), Milk–Inulin (50% *w*/*w* skimmed milk with 5.0% *w*/*w* inulin), Yogurt (50% *w*/*w* plain drinking yogurt), and Yogurt–-Inulin (50% *w*/*w* plain drinking yogurt with 5.0% *w*/*w* inulin). Only male rats were included to reduce animal-to-animal variation and thereby increase statistical power. The rats were fed the diets for 6 weeks. Then, at study termination, the rats were sacrificed by bleeding under fentanyl/fluanison/midazolam anesthesia (Hypnorm, Skanderborg Apotek, Denmark, and Midazolam 5 mg/mL, Braun, Frederiksberg, Denmark) and after dissection, CMS and liver samples (approx. 300 mg) were collected and stored at −80 °C. The left femur bones were dissected free and stored at −20 °C for further analyses.

### 2.2. RNA Extraction and Transcriptome Analysis

Prior to RNA extraction, the femur bone was processed according to a procedure described by Nance et al. [17] with minor modifications. In brief, the diaphysis was obtained after the removal of epiphyses using a shearing cutter and of bone marrow by centrifugation at 10,000× *g* for 10 min at room temperature. Then, the clean diaphysis was grounded using RNAse-free mortar and pestle with liquid nitrogen inside to finally obtain crushed bone powder.

Total RNA was extracted from approx. 10 mg of colonic mucus scrabs, 15 mg of liver tissue, and 25 mg of femur powder, respectively, using Trizol following the manufacturer’s protocol (Sigma-Aldrich, St. Louis, MO, USA). Hereafter, transcriptome profiling was implemented by the Affymetrx Clariom S mouse as described in a previous study [18]. In brief, cRNA was synthesized from 100 ng of total RNA using the GeneChip WT Plus Reagent Kit (Thermo Fisher Scientific Inc., Waltham, MA, USA) and processed according to the manufacturer’s protocol. After fragmentation and labeling, the samples were loaded onto arrays for hybridization over 16 h. Subsequently, the arrays were washed using the Fluidic Station 450s (Thermo Fisher Scientific Inc., Waltham, MA, USA) and scanned with the GeneChip Scanner (Thermo Fisher Scientific Inc., Waltham, MA, USA). The resulting data were analyzed using the Transcriptome Analysis Console software 4.0.2 (Applied Biosystems, Thermo Fisher Scientific Inc., Waltham, MA, USA) with an FDR threshold of <0.1. The transcriptome of colonic mucus scrubs, liver, and bone were all compiled on a total of 12 rats randomly selected from the four intervention groups: Milk (*n* = 3), Milk–Inulin (*n* = 3), Yogurt (*n* = 3), Yogurt–Inulin (*n* = 3). The analyses were limited to samples from three animals per treatment group as a result of a lack of resources for additional GeneChips.

GO and KEGG pathways were analyzed through an online tool named G: profiler (https://biit.cs.ut.ee/gprofiler/gost, accessed on 25 October 2023).

### 2.3. qPCR

An amount of 800 ng extracted RNA was subjected to reverse transcriptase into cDNA using the iScript kit (Bio-Rad, California, USA) according to the manufacturer protocol. qPCR Analyses were compiled on a total of 16 rats from the two intervention groups: Milk (*n* = 8) and Yogurt (*n* = 8).

To evaluate the specific content of mRNA of selected genes in CMS, real-time PCR was performed using TaqMan probes as previously described [19]. Rat-specific primers and probes pairs were designed following the method described by Rasmussen et al. [20]. The used primers and TaqMan probe sequences are shown in Table 1.

The relative mRNA content was calculated from the obtained Ct-values and normalized to the content of Eef1a1. The mRNA content of the housekeeping gene Eef1a1 was not significantly different between groups.

### 2.4. Statistical Analysis

All data were presented as mean ± standard error of the mean (SEM). Statically significant differences between groups were evaluated by one-way ANOVA with Tukey’s post hoc test for real-time PCR statistical analysis (Origin Pro 2018, OriginLab Corp., Northampton, MA, USA). If the equal variance test failed, data were log10 transformed before executing the ANOVA.

Spearman’s correlation analysis (*n* = 10) was performed between DEGs and microbial taxa relative abundance from the CMS transcriptome and intestinal microbiome [16] using GraphPad Prism 10.4.1 (La Jolla, CA, USA).

## 3. Results

### 3.1. Distinct Transcriptome Features of Different Tissue Types

**Figure 1:**
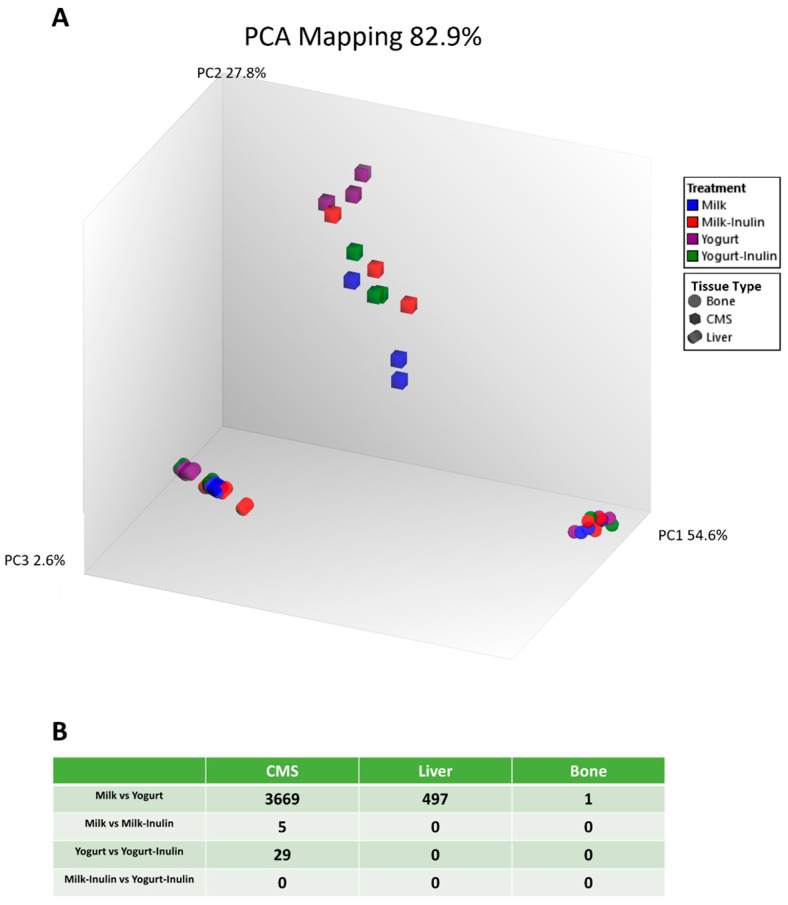
Transcriptomic profiling of CMS, liver, and bone tissue after a 6-week intervention with 4 different diets: Milk, Milk–Inulin, Yogurt, and Yogurt–Inulin. (**A**) PCA mapping of three tissues/organs with principal component (PC1), PC2, and PC3 explaining 54.6%, 27.8%, and 2.6% of the total variation, respectively. (**B**) The number of DEGs across groups and tissue types with a *q*-value of <0.10.

Principal component analysis (PCA) based on transcriptome data for all samples showed a clear clustering of the three tissue types (CMS, liver, and bone) (Figure 1A). A separation of the different diet groups was evident within the CMS samples, and to a minor extent also evident for liver samples, while bone samples were so close in position in the score plot that a diet separation was not evident. Consequently, the PCA score plot revealed that CMS samples were most responsive to diet-induced changes in the transcriptome while bone samples were least responsive to diet-induced changes. Differentially expressed genes (DEGs) between dietary groups were identified in three organs with *q*-value < 0.10 and log_2_(fold change) > 2 or <−2. The number of DEGs between Milk and Yogurt was 3669 in CMS, 497 in liver, and 1 in bone. A total of 5 DEGs were identified between Milk and Milk–Inulin, 29 DEGs were found between Yogurt and Yogurt–Inulin in CMS, while no DEGs were identified in liver and in bone when comparing Inulin with non-inulin groups (Figure 1B).

### 3.2. The CMS Transcriptome

**Figure 2:**
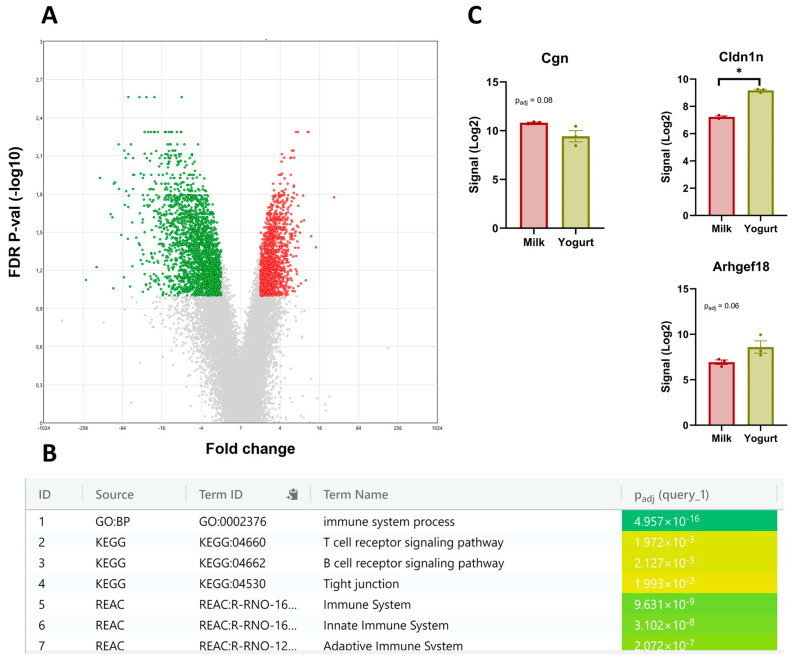
CMS transcriptomics after a 6-week diet intervention where the Milk and the Yogurt groups are compared. (**A**) Volcano plot shows 3669 DEGs with significant upregulated (green) and downregulated (red) genes highlighted. A *q*-value of <0.10 was used to determine significance. (**B**) Pathway enrichment analysis performed on the top upregulated genes. (**C**). Signal intensities of *Cldnd1*, *Arhgef18*, and *Cgn*. Reported values are LSMeans and bars indicate standard errors. The symbol * denotes significance at the level P_adj_ < 0.05.

A comparison of the Milk and the Yogurt group revealed 2195 upregulated and 1474 downregulated DEGs in CMS (Figure 2A). To elucidate the functional roles and significant pathways of DEGs between the Milk and the Yogurt group, the most highly upregulated and downregulated genes were analyzed through G: Profiler for GO categories and KEGG pathway analyses. The analysis of the top upregulated DEGs displayed enriched pathways for tight junction and the immune system (Figure 2B). From the DEG pool, we identified three genes involved in tight junction, of which two of the genes were upregulated (*Cldnd1*, *Arhgef18*), while one gene was downregulated (*Cgn*) in Yogurt (Figure 2C).

**Figure 3:**
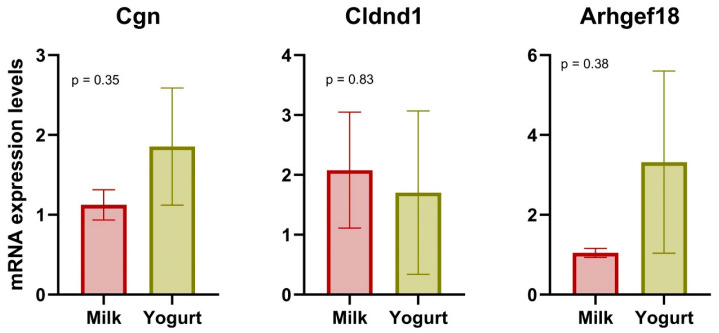
Relative mRNA content in CMS for the Milk (*n* = 8) and the Yogurt (*n* = 8) groups after a 6-week diet intervention.

To support the transcriptomic analyses, real-time PCR was performed on three selected genes linked to tight junction function (Figure 3). While the results were not statistically significant, the mRNA expression level of *Arhgef18* was higher for the Yogurt group, consistent with transcriptomic analysis.

Associations between the three DEGs *Clnd1, Arhgef18,* and *Cgn* and an abundance of bacterial species from the intestine were elucidated by Spearman’s correlation analysis and the top 15 highest correlations for each gene are presented in Table S1. Genus of *Clostridium* was negatively correlated and *Blautia* and *Bifidobacterium_pseudolongum* were positively correlated with the expression levels of *Arghef18* and *Clnd1*. In addition, *Lactobacillus_murinus* were found positively correlated to the expression level of *Cgn* while negatively correlated with that of Arghef18.

No significant difference in pathway enrichment was found when comparing Milk vs. Milk–Inulin, Yogurt vs. Yogurt–Inulin, and Milk–Inulin vs. Yogurt–Inulin in CMS because of the low number of DEGs between these paired groups (Figure 1B).

### 3.3. The Liver Transcriptome

**Figure 4:**
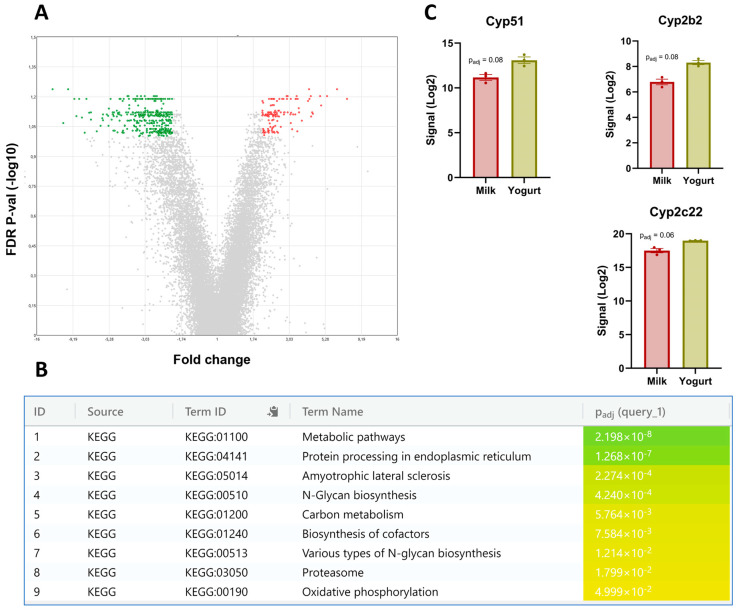
Liver transcriptomics after a 6-week diet intervention where the Milk and the Yogurt groups are compared. (**A**) Volcano plot shows 497 DEGs with significant upregulated (green) and downregulated (red) genes highlighted. A *q*-value of <0.10 was used to determine significance. (**B**) Pathway enrichment analysis performed on the top upregulated genes. (**C**) Signal intensities of *Cyp51*, *Cyp2b2*, and *Cyp2c22*. Reported values are LSMeans and bars indicate standard errors.

Comparison of Milk and Yogurt revealed that 370 DEGs were upregulated and 127 DEGs were downregulated for Yogurt compared with Milk (Figure 4A). Results from KEGG pathway analyses further revealed that metabolic pathways were top enriched in the Yogurt (Figure 4B) with *Cyp51*, *Cyp2b2*, and *Cyp2c22* being upregulated (Figure 4C).

There were no DEGs identified when comparing Milk vs. Milk–Inulin, Yogurt vs. Yogurt–Inulin, and Milk–Inulin vs. Yogurt–Inulin in the liver.

### 3.4. The Bone Transcriptome

Except one DEG in the comparison of milk and yogurt, there were no DEGs identified across comparisons of the diet combinations in the transcriptomic profiling of bone (Figure 1B).

## 4. Discussion

Fermented dairy products are attracting scientific interest due to their health-promoting effects. It has been proposed that yogurt improves immune function through its direct and indirect impact on the gut, which leads to shifts in circulating and systemic biomarkers and triggers alterations in other tissues [21]. Similarly, yogurt consumption has been suggested to maintain microbial balance and reduce the risk of cardiometabolic disease [22]. However, currently, limited mechanistic evidence is available, and it is therefore essential to further investigate the molecular mechanisms by which the consumption of fermented dairy products exerts unique effects on the gut as well as gut–organ axes. This rat study was conducted to compare fermented with non-fermented dairy products alone or fortified with inulin in the way of their influences on the gut, liver, and bone using transcriptomics as the main tool.

### 4.1. Tissue Type

Transcriptomic features of the three tissue types investigated were distinguished by PCA, showing tissue/organ-specific responses to the diet intervention. The largest number of DEGs was identified in CMS (3669), while 497 DEGs and 1 DEG were identified in the liver and femur, respectively, when comparing Milk vs. Yogurt. This finding can be explained by the direct influence of food on the mucosa tissue in the gastrointestinal tract. Dietary constituents are broken down into small molecules and absorbed in the gastrointestinal tract during which the gut environment tends to be changed directly [23]. Subsequently, these changes in the gut environment induced by the diet may indirectly lead to alterations in other organs including the liver function and bone metabolism coherent with the existence of a gut–organ axis [15].

### 4.2. Gene Expression in CMS

Differences in CMS between the Milk and Yogurt groups were observed at the gene level. Three genes linked to tight junction function were upregulated (*Cldnd1* [24], *Arhgef18* [25]) or downregulated (*Cgn* [26]) in rats receiving the yogurt-based diet. According to GO categories, a tight junction-related pathway was also enriched for the Yogurt group. However, q-PCR results showed no significant difference in these genes between the diets. Tight junctions are composed of transmembrane proteins and serve as the primary barrier to intestinal intercellular space [27]. Tight junctions might be targeted by bacteria, resulting in their disruption by pathogens or enhanced synthesis in the presence of probiotics [28,29,30]. Thus, genera such as *Blautia* and *Bifidobacterium_pseudolongum* were reported to be positively associated with tight junction integrity, whereas *Clostridium* showed a negative correlation [31,32,33]. Consistently, Spearman’s correlation analysis conducted in the present study showed resembling relations between these genera and tight junction-related DEGs. In some previous studies, the possibility that yogurt consumption could enhance the expression of tight junction proteins has been reported [34,35,36]. This effect could be explained by the presence of lactic acid bacteria in yogurt, which exhibits the potential to strengthen these adhesion protein complexes [29,37]. Even though non-significant, our study also indicated an effect of yogurt consumption on the gene expression of these tight junction proteins, and our previous 16S sequencing result on fecal samples verified the higher abundance of lactobicillales in the Yogurt than that in the Milk group [16]. Tight junctions are essential to the integrity of epithelial and endothelial barriers. A reduced barrier function and an increased intestinal permeability is associated with many intestinal and systemic diseases including metabolic syndrome and type 2 diabetes [38]. The present findings indicating that yogurt consumption influences the regulation of tight junction genes could indicate that this is a potential mechanism involved in the reduced risk of metabolic syndrome and type 2 diabetes development that has been reported for yogurt in cohort studies [39].

In addition to the potentially beneficial effects on tight junction functions, the lactic acid bacteria contained in fermented dairy have also been proposed to be associated with stimulating and regulating immune responses by modifying the intestinal environment [27]. Consistently, the current study found that pathways related to the immune system were enriched with yogurt consumption as revealed by KEGG pathway analysis. Despite that KEGG pathway analysis revealed the effects of yogurt consumption on pathways related to the immune system, this was not reflected in effects on serum levels of the inflammatory markers TNF-α and IL-6, which we analyzed in a former study [16]. Serum levels of TNF-α and IL-6 levels were found to be low, probably because the rats were young and, in general, healthy [16].

### 4.3. Gene Expression in Liver

Transcriptomic profiling of liver tissue revealed that a total of 370 genes were upregulated in the Yogurt group compared to the Milk group; among these, *Cyp51* [40], *Cyp2c22* [41], *Cyp2b2* [42] are the cytochrome p450s involved in hepatic function in (modified) drug metabolism [18]. In addition, KEGG pathway analysis also characterized metabolic pathways being enriched with yogurt consumption. The liver is an important metabolic organ regulating whole-body homeostasis [43], and our findings suggest that its function is influenced by the intake of fermented dairy, and it supports the existence of a gut–liver axis [44].

### 4.4. Gene Expression in Bone

In addition, the intake of dairy products has also been proposed to exert beneficial effects on bone mineralization and bone health [45,46]. Considering that prebiotics and fermented dairy products may alter the gut microbiota and promote calcium translocation across the intestinal epithelium [47], we expected that yogurt with/without inulin addition may exert a distinctly beneficial effect on bone transcriptome compared to milk consumption with/without inulin supplementation. However, our results showed no evident differences in the bone transcriptome between the diet groups, indicating that any potential effects of fermented dairy product consumption on bone metabolism did not involve gene regulation.

### 4.5. Inulin Effects

Several former studies have demonstrated that inulin supplementation has the potential to impact the liver transcriptome [14,48,49], while the effects on the transcriptome of intestinal tissue have only been sparsely documented [50]. The present study found that inulin supplementation had a minor effect on the tissue/organ transcriptome when compared to the effect of intake of a fermented dairy product. This finding that the intake of fermented dairy has a much more pronounced effect on endogenous processes involving the regulation of gene expression than supplementation with prebiotics indicates that the presence of a live starter culture is crucial for obtaining gene-regulating effects in the intestinal tissue. While former studies have revealed pronounced effects of the administration of probiotics including lactobacilli on gene expression in human mucosa [51,52], to the best of our knowledge, the present study is the first to demonstrate how intake of fermented dairy has a resembling strong potential to modulate gene expression in the gastrointestinal tract. 

### 4.6. Study Limitations

The present study had some limitations. Only male rats were included, and whether there are sex-specific variations in the responses remain unknown. Sex hormones may modulate gut–organ interactions and if possible, future studies should preferably include both sexes for broader pertinence. In addition, the transcriptomics analyses were conducted on only three samples per treatment group, which can be ascribed to the cost of the analyses. The limited number of samples reduces the statistical power and lowers confidence in the robustness of differentially expressed genes (DEGs) identified and the pathway enrichment result. The gene expression analyses were not corroborated by analysis of protein expression. Consequently, while the present findings demonstrated marked effects of food matrix on intestinal gene expression, further studies on larger sample sets to elaborate and confirm the present findings at both gene and protein levels are warranted.

## 5. Conclusions

The current study revealed that consumption of fermented dairy resulted in pronounced effects on the transcriptome of colon mucosa of healthy rats when compared with consumption of a corresponding non-fermented dairy product. The effect of inulin fortification of diets had less pronounced effects on the transcriptome of the colon mucosa, indicating that intake of fermented dairy exerts different effects on gene expression in the intestinal tissue than intake of prebiotics. The study also demonstrated that colon mucosa was the tissue type under investigation that was mostly affected by the food matrix, but the regulation of gene expression was also evident in the liver, supporting that a diet-induced gut–liver axis also acts on the gene level, while this could not be demonstrated for bone tissue.

## Figures and Tables

**Table 1:** Primer and TaqMan probes for real-time PCR.

Name	Forward (5′-3′)	Reverse (5′-3′)	TaqMan Probe (5′-3′)
Cgn	CCTGCCTTGCTGCTTTACTC	GGCTGGTCTTTGGAGAGGTATG	TCAGTGCCTTACCAGTGTGCGA
Arhgef18	GAATGCTGAGTCTGTTTTCATAGAAGA	CCTCAAACTCATGGGCATCTG	TGAGGTGTGAGATCGAA
Cldnd1	CGTGACTGCTCAGGCCATCT	CGGTGCTTTGCGAAACG	AGGGACTGTGGATGTC
Eeflal	AGCAAAAATGACCCACCAATG	GATCTGGCCTGGATGGTTCA	CAGCTGGCTTCACTGCTCAGGTGATTATC

Cgn, cingulin; Flna, filamin A; Arhgef18, Rho/Rac guanine nucleotide exchange factor 18; Cldnd1, claudin domain containing 1; Eeflal, Eukaryotic Elongation Factor 1A1.

## Data Availability

Data available in a publicly accessible repository: “Effects of dairy matrix on the intestinal, liver, and bone transcriptome of healthy rats”, doi:10.17632/nt7v283j9x.1.

## Supplementary Materials

The following supporting information can be downloaded at https://www.mdpi.com/article/10.3390/foods14081375/s1. Table S1: Spearman’s correlation analysis between differentially expressed gene (DEGs) and microbial taxa relative abundance.

## Abbreviations

The following abbreviations are used in this manuscript:

CMS	Colon mucosa scrubs
DEG	Differential expressed genes
GO	Gene ontology
KEGG	Kyoto encyclopedia of genes and genomes
PCA	Principal component analysis
