# Supplementary material for: Effects of Dairy Matrix on the Intestinal, Liver, and Bone Transcriptome of Healthy Rats
Source: Foods. 2025 Apr 16;14(8):1375. doi: 10.3390/foods14081375 (PMC12027020; doi:10.3390/foods14081375)
Supplement: Supplementary file 1 [file foods-14-01375-s001.zip › foods-3553036-supplementary.pdf]

Table S1. Spearman's correlation analysis between differentially expressed gene (DEGs) and microbial taxa relative abundance. The table includes top 15 bacterial species exhibiting the highest correlation coefficients with each DEG.

**Spearman R-value**	**P-value**	**Bacterium species**
**Cgn**		
-0.9004	0.0009	*Parabacteroides*
-0.8997	0.0009	*Parabacteroides*
-0.8415	0.0036	*Muribaculaceae;g__;s__uncultured_bacterium*
-0.8273	0.0049	*Muribaculaceae;g__;s__uncultured_bacterium*
-0.8262	0.0056	*Streptococcus;s__uncultured_bacterium*
-0.8241	0.0079	*Muribaculaceae;g__;s__uncultured_bacterium*
-0.816	0.0064	*Muribaculaceae;g__;s__uncultured_bacterium*
0.809	0.0056	*Bifidobacterium_pseudolongum*
-0.8061	0.0072	*Muribaculaceae;g__;s__uncultured_bacterium*
0.803	0.008	*Lactobacillus_murinus*
-0.7979	0.0083	*Lactobacillus_paracasei*
-0.7939	0.0088	*Muribaculaceae;g__;s__uncultured_bacterium*
-0.7866	0.0097	*Muribaculaceae;g__;s__uncultured_bacterium*
-0.7823	0.012	*Muribaculaceae;g__;s__uncultured_bacterium*
-0.7693	0.0148	*Muribaculaceae;g__;s__uncultured_bacterium*
**Arghef18**		
0.8495	0.0033	*Blautia*
0.8492	0.003	*Bifidobacterium_pseudolongum*
0.8492	0.003	*Muribaculaceae;g__;s__uncultured_bacterium*
0.8365	0.0045	*Lachnospiraceae_UCG-008;s__uncultured_bacterium*
0.8355	0.004	*Bifidobacterium_pseudolongum*
0.8272	0.0044	*Muribaculaceae;g__;s__uncultured_bacterium*
0.8113	0.0075	*Lachnospiraceae*
0.8102	0.0058	*Muribaculaceae;g__;s__uncultured_bacterium*
0.8081	0.0077	*Blautia;s__uncultured_bacterium*
0.8081	0.0077	*Blautia*
-0.8079	0.0064	*Lactobacillus_murinus*
0.8044	0.0095	*Blautia;s__uncultured_bacterium*
0.7976	0.0092	*Lachnospiraceae;g__GCA-900066575;s__uncultured_bacterium*
0.7964	0.0079	*Muribaculaceae;g__;s__uncultured_bacterium*
0.7944	0.0097	*Bifidobacterium;s__Bifidobacterium_pseudolongum*
**Clnd1**		
-0.9442	0.0002	*Clostridium_sensu_stricto_1;s__uncultured_bacterium*
-0.8774	0.0018	*Lachnospiraceae;g__uncultured;s__uncultured_bacterium*
0.8636	0.0024	*Lachnospiraceae;g__GCA-900066575;s__uncultured_bacterium*
-0.8398	0.004	*Bacillus*
-0.8398	0.004	*Bacilli*
-0.8398	0.004	*Clostridium_sensu_stricto_1;s__uncultured_bacterium*
-0.8398	0.004	*Turicibacter;s__uncultured_bacterium*
-0.8398	0.004	*Clostridium_sensu_stricto_1;s__uncultured_bacterium*
0.833	0.0048	*Turicibacter;s__uncultured_bacterium*
-0.8283	0.0052	*Lachnospiraceae_NK4A136_group;s__uncultured_bacterium*
0.8157	0.0071	*Blautia;s__uncultured_bacterium*
-0.8128	0.0028	*Clostridium_sensu_stricto_1;s__uncultured_bacterium*
-0.8128	0.0028	*Clostridium_sensu_stricto_1;s__uncultured_bacterium*
-0.8128	0.0028	*Clostridium_sensu_stricto_1;s__uncultured_bacterium*

0.8081	0.0083	*Blautia*
